# Tetra­aqua­bis­[4-(4*H*-1,2,4-triazol-4-yl)benzoato-κ*N* ^1^]copper(II) dihydrate

**DOI:** 10.1107/S1600536811018356

**Published:** 2011-05-20

**Authors:** Shuzhi Xu, Wenxin Shao, Miao Yu, Guihua Gong

**Affiliations:** a Jilin Business and Technology College, Changchun 130062, People’s Republic of China

## Abstract

In the title compound, [Cu(C_9_H_6_N_3_O_2_)_2_(H_2_O)_4_]·2H_2_O, the Cu^II^ atom lies on an inversion center and is six-coordinated by two N atoms from two 4-(1,2,4-triazol-4-yl)benzoate ligands and four water mol­ecules in a distorted octa­hedral geometry. In the crystal, inter­molecular O—H⋯O hydrogen bonds lead to a three-dimensional supra­molecular network. Intra­molecular O—H⋯N hydrogen bonds and π–π inter­actions between the benzene rings and between the benzene and triazole rings [centroid–centroid distances = 3.657 (1) and 3.752 (1) Å] are observed.

## Related literature

For general background to the structures and applications of inorganic–organic hybrid materials, see: Shi *et al.* (2009 ▶); Xiao *et al.* (2006 ▶); Zhang *et al.* (2004 ▶). For a related structure, see: Wang *et al.* (2009 ▶).
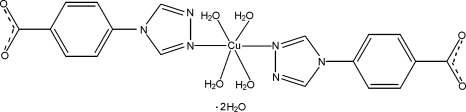


## Experimental

### 

#### Crystal data

[Cu(C_9_H_6_N_3_O_2_)_2_(H_2_O)_4_]·2H_2_O
*M* *_r_* = 547.97
Triclinic, 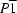

*a* = 7.3001 (4) Å
*b* = 7.9904 (5) Å
*c* = 9.8995 (6) Å
α = 85.343 (1)°
β = 73.243 (1)°
γ = 79.032 (1)°
*V* = 542.61 (6) Å^3^
*Z* = 1
Mo *K*α radiation
μ = 1.08 mm^−1^
*T* = 293 K
0.24 × 0.22 × 0.19 mm

#### Data collection

Bruker APEXII CCD diffractometer
Absorption correction: multi-scan (*SADABS*; Bruker, 2001 ▶) *T*_min_ = 0.75, *T*_max_ = 0.83
3001 measured reflections
2102 independent reflections
2025 reflections with *I* > 2σ(*I*)
*R*_int_ = 0.033

#### Refinement

*R*[*F* ^2^ > 2σ(*F* ^2^)] = 0.034
*wR*(*F* ^2^) = 0.091
*S* = 1.12
2102 reflections
178 parameters
7 restraints
H atoms treated by a mixture of independent and constrained refinement
Δρ_max_ = 0.81 e Å^−3^
Δρ_min_ = −0.69 e Å^−3^

### 

Data collection: *APEX2* (Bruker, 2007 ▶); cell refinement: *SAINT* (Bruker, 2007 ▶); data reduction: *SAINT*; program(s) used to solve structure: *SHELXTL* (Sheldrick, 2008 ▶); program(s) used to refine structure: *SHELXTL*; molecular graphics: *SHELXTL* and *Materials­Studio* (Accelrys, 2006 ▶); software used to prepare material for publication: *SHELXTL*.

## Supplementary Material

Crystal structure: contains datablocks global, I. DOI: 10.1107/S1600536811018356/hy2429sup1.cif

Structure factors: contains datablocks I. DOI: 10.1107/S1600536811018356/hy2429Isup2.hkl

Additional supplementary materials:  crystallographic information; 3D view; checkCIF report

## Figures and Tables

**Table 1:** Selected bond lengths (Å)

Cu1—O1*W*	1.9937 (19)
Cu1—O2*W*	2.4932 (16)
Cu1—N2	2.0535 (18)

**Table 2:** Hydrogen-bond geometry (Å, °)

*D*—H⋯*A*	*D*—H	H⋯*A*	*D*⋯*A*	*D*—H⋯*A*
O1*W*—H1*A*⋯O2^i^	0.87 (2)	2.12 (2)	2.948 (3)	158 (2)
O1*W*—H1*B*⋯N3^ii^	0.87 (2)	2.27 (3)	2.873 (3)	126 (3)
O2*W*—H2*A*⋯O1^iii^	0.82 (3)	1.98 (3)	2.794 (2)	172 (3)
O2*W*—H2*B*⋯O2^iv^	0.82 (3)	1.91 (3)	2.711 (2)	167 (3)
O3*W*—H3*A*⋯O2^i^	0.84 (3)	1.97 (3)	2.789 (2)	167 (3)
O3*W*—H3*B*⋯O2*W*^v^	0.82 (3)	1.94 (3)	2.758 (2)	170 (3)

Symmetry codes: (i) 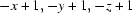; (ii) 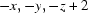; (iii) 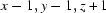; (iv) 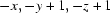; (v) 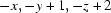.

**Fig. 1.**
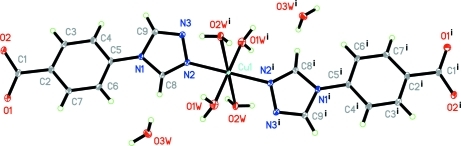
The molecular structure of the title compound. Displacement ellipsoids are drawn at the 30% probability level. [Symmetry code: (i) -x, -y, 2-z.]

**Fig. 2.**
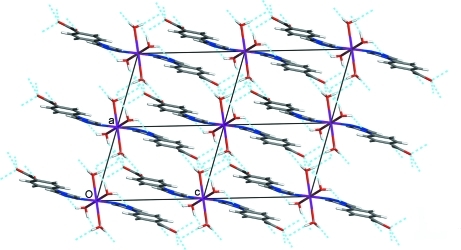
View of the three-dimensional network of the title compound, built by hydrogen bonds (dashed lines).

## supplementary crystallographic information

### Comment

There has been considerable interest in inorganic–organic hybrid materials with variable dimensionality and different coordination frameworks (Shi *et al.*, 2009; Xiao *et al.*, 2006). The studies on these inorganic–organic hybrid pillared structures focus on aspects concerning materials science and structural chemistry because of their potential applications in catalysis, sorption processes, photochemistry and magnetism (Zhang *et al.*, 2004). In this contribution, we selected 4-(1,2,4-triazol-4-yl)benzoic acid (Htyb) as an organic carboxylate ligand, generating the title coordination compound, which is reported here.

In the title compound, the Cu^II^ ion, lying on an inversion center, is six-coordinated by two N atoms from two tyb ligands and four water molecules and shows a distorted octahedral coordination geometry (Fig. 1). Two uncoordinated water molecules exist in the structure, stabilized by hydrogen bonds (Table 1). The Cu—N and Cu—O bond lengths and the O—Cu—O and N—Cu—O bond angles are comparable to those found in other Cu(II) complexes (Wang *et al.*, 2009).  In the crystal, intermolecular O—H···O hydrogen bonds (Table 2) lead to a three-dimensional supramolecular network (Fig. 2). Intramolecular O—H···N hydrogen bonds, as well as π–π interactions, *Cg*1···*Cg*1^ii^ = 3.657 (1) and *Cg*1···*Cg*2^iv^ = 3.752 (1) Å [*Cg*1 and *Cg*2 are the centroids of C2–C7 ring and N1–N3, C8, C9 ring. Symmetry codes: (ii) 1-x, 1-y, 1-z; (iv) -x, 1-y, 1-z], are observed.

### Experimental

A mixture of 4-(1,2,4-triazol-4-yl)benzoic acid (0.4 mmol, 0.075 g) and NaOH (0.4 mmol, 0.016 g) in water (15 ml) was added with CuCl_2_.2H_2_O (0.2 mmol, 0.034 g), giving a blue precipitate. The precipitate was dissolved by dropwise addition of diluted ammonia. Blue crystals were obtained from the filtrate by slow evaporation after standing for several days.

### Refinement

H atoms on C atoms were positioned geometrically and refined as riding atoms, with C—H = 0.93 Å and *U*_iso_(H) = 1.2*U*_eq_(C). H atoms of water molecules were located in a difference Fourier map and refined with O—H distance restraints of 0.85 (1) Å and with *U*_iso_(H) = 1.5*U*_eq_(O).

### Crystal data

**Table:**

[Cu(C_9_H_6_N_3_O_2_)_2_(H_2_O)_4_]·2H_2_O	*Z* = 1
*M**_r_* = 547.97	*F*(000) = 283
Triclinic, *P*1	*D*_x_ = 1.677 Mg m^−^^3^
Hall symbol: -P 1	Mo *K*α radiation, λ = 0.71073 Å
*a* = 7.3001 (4) Å	Cell parameters from 2131 reflections
*b* = 7.9904 (5) Å	θ = 1.0–26.0°
*c* = 9.8995 (6) Å	µ = 1.08 mm^−^^1^
α = 85.343 (1)°	*T* = 293 K
β = 73.243 (1)°	Block, blue
γ = 79.032 (1)°	0.24 × 0.22 × 0.19 mm
*V* = 542.61 (6) Å^3^	

### Data collection

**Table:**

Bruker APEXII CCD diffractometer	2102 independent reflections
Radiation source: fine-focus sealed tube	2025 reflections with *I* > 2σ(*I*)
graphite	*R*_int_ = 0.033
φ and ω scans	θ_max_ = 26.0°, θ_min_ = 2.2°
Absorption correction: multi-scan (*SADABS*; Bruker, 2001)	*h* = −7→9
*T*_min_ = 0.75, *T*_max_ = 0.83	*k* = −9→9
3001 measured reflections	*l* = −10→12

### Refinement

**Table:**

Refinement on *F*^2^	Primary atom site location: structure-invariant direct methods
Least-squares matrix: full	Secondary atom site location: difference Fourier map
*R*[*F*^2^ > 2σ(*F*^2^)] = 0.034	Hydrogen site location: inferred from neighbouring sites
*wR*(*F*^2^) = 0.091	H atoms treated by a mixture of independent and constrained refinement
*S* = 1.12	*w* = 1/[σ^2^(*F*_o_^2^) + (0.048*P*)^2^ + 0.4256*P*] where *P* = (*F*_o_^2^ + 2*F*_c_^2^)/3
2102 reflections	(Δ/σ)_max_ < 0.001
178 parameters	Δρ_max_ = 0.81 e Å^−^^3^
7 restraints	Δρ_min_ = −0.69 e Å^−^^3^

### Fractional atomic coordinates and isotropic or equivalent isotropic displacement parameters (Å^2^)

**Table:**

	*x*	*y*	*z*	*U*_iso_*/*U*_eq_	
Cu1	0.0000	0.0000	1.0000	0.01356 (14)	
C1	0.4338 (3)	0.8158 (3)	0.2433 (2)	0.0171 (4)	
C2	0.3525 (3)	0.6774 (3)	0.3431 (2)	0.0159 (4)	
C3	0.3242 (3)	0.5307 (3)	0.2906 (2)	0.0173 (4)	
H3	0.3500	0.5208	0.1937	0.021*	
C4	0.2582 (3)	0.3996 (3)	0.3803 (2)	0.0177 (4)	
H4	0.2397	0.3021	0.3442	0.021*	
C5	0.2198 (3)	0.4155 (3)	0.5251 (2)	0.0144 (4)	
C6	0.2448 (3)	0.5605 (3)	0.5801 (2)	0.0170 (4)	
H6	0.2177	0.5704	0.6771	0.020*	
C7	0.3109 (3)	0.6909 (3)	0.4887 (2)	0.0169 (4)	
H7	0.3277	0.7889	0.5251	0.020*	
C8	0.0901 (3)	0.2734 (3)	0.7605 (2)	0.0158 (4)	
H8	0.0776	0.3639	0.8178	0.019*	
C9	0.1476 (4)	0.1191 (3)	0.5795 (2)	0.0239 (5)	
H9	0.1839	0.0838	0.4868	0.029*	
N1	0.1555 (3)	0.2774 (2)	0.61779 (19)	0.0150 (4)	
N2	0.0469 (3)	0.1231 (2)	0.80595 (18)	0.0157 (4)	
N3	0.0828 (3)	0.0245 (2)	0.6893 (2)	0.0242 (4)	
O1	0.4190 (3)	0.9593 (2)	0.29038 (18)	0.0286 (4)	
O2	0.5161 (2)	0.7736 (2)	0.11668 (16)	0.0189 (3)	
O1W	0.1051 (3)	0.1744 (3)	1.0745 (2)	0.0310 (4)	
H1A	0.2286 (18)	0.1646 (15)	1.030 (3)	0.047*	
H1B	0.107 (5)	0.146 (5)	1.1613 (17)	0.047*	
O2W	−0.3408 (2)	0.1514 (2)	1.09311 (17)	0.0201 (3)	
H2A	−0.402 (4)	0.089 (4)	1.152 (3)	0.030*	
H2B	−0.384 (4)	0.159 (4)	1.025 (3)	0.030*	
O3W	0.2262 (3)	0.5346 (2)	0.94116 (19)	0.0244 (4)	
H3A	0.316 (4)	0.450 (3)	0.927 (4)	0.037*	
H3B	0.270 (5)	0.624 (3)	0.922 (3)	0.037*	

### Atomic displacement parameters (Å^2^)

**Table:**

	*U*^11^	*U*^22^	*U*^33^	*U*^12^	*U*^13^	*U*^23^
Cu1	0.0179 (2)	0.0127 (2)	0.0086 (2)	−0.00537 (14)	−0.00024 (14)	0.00153 (13)
C1	0.0141 (10)	0.0192 (11)	0.0151 (10)	−0.0029 (8)	−0.0005 (8)	0.0039 (8)
C2	0.0125 (10)	0.0170 (10)	0.0152 (10)	−0.0019 (8)	−0.0004 (8)	0.0036 (8)
C3	0.0167 (10)	0.0232 (11)	0.0106 (10)	−0.0043 (8)	−0.0016 (8)	0.0008 (8)
C4	0.0188 (11)	0.0197 (11)	0.0147 (10)	−0.0074 (9)	−0.0025 (8)	0.0009 (8)
C5	0.0122 (10)	0.0158 (10)	0.0124 (10)	−0.0023 (8)	−0.0006 (8)	0.0046 (8)
C6	0.0183 (10)	0.0174 (10)	0.0114 (10)	−0.0007 (8)	0.0000 (8)	0.0005 (8)
C7	0.0177 (10)	0.0148 (10)	0.0151 (10)	−0.0018 (8)	−0.0006 (8)	−0.0005 (8)
C8	0.0183 (10)	0.0159 (10)	0.0113 (10)	−0.0039 (8)	−0.0012 (8)	0.0017 (8)
C9	0.0384 (14)	0.0164 (11)	0.0125 (11)	−0.0065 (10)	0.0009 (9)	−0.0004 (8)
N1	0.0163 (9)	0.0143 (9)	0.0115 (8)	−0.0034 (7)	0.0005 (7)	0.0018 (7)
N2	0.0180 (9)	0.0156 (9)	0.0112 (8)	−0.0039 (7)	−0.0002 (7)	0.0001 (7)
N3	0.0397 (12)	0.0175 (10)	0.0119 (9)	−0.0086 (9)	0.0009 (8)	−0.0015 (7)
O1	0.0405 (10)	0.0178 (9)	0.0202 (9)	−0.0109 (7)	0.0064 (7)	−0.0005 (7)
O2	0.0203 (8)	0.0193 (8)	0.0139 (8)	−0.0057 (6)	0.0007 (6)	0.0030 (6)
O1W	0.0343 (10)	0.0347 (10)	0.0231 (9)	−0.0126 (8)	−0.0029 (8)	0.0019 (8)
O2W	0.0225 (9)	0.0205 (8)	0.0166 (8)	−0.0076 (7)	−0.0032 (7)	0.0045 (6)
O3W	0.0219 (9)	0.0178 (8)	0.0303 (9)	−0.0047 (7)	−0.0005 (7)	−0.0028 (7)

### Geometric parameters (Å, °)

**Table:**

Cu1—O1W	1.9937 (19)	C6—H6	0.9300
Cu1—O2W	2.4932 (16)	C7—H7	0.9300
Cu1—N2	2.0535 (18)	C8—N2	1.311 (3)
C1—O1	1.246 (3)	C8—N1	1.354 (3)
C1—O2	1.266 (3)	C8—H8	0.9300
C1—C2	1.512 (3)	C9—N3	1.297 (3)
C2—C3	1.393 (3)	C9—N1	1.366 (3)
C2—C7	1.393 (3)	C9—H9	0.9300
C3—C4	1.383 (3)	N2—N3	1.385 (3)
C3—H3	0.9300	O1W—H1A	0.87 (2)
C4—C5	1.391 (3)	O1W—H1B	0.87 (2)
C4—H4	0.9300	O2W—H2A	0.82 (3)
C5—C6	1.383 (3)	O2W—H2B	0.82 (3)
C5—N1	1.437 (3)	O3W—H3A	0.84 (3)
C6—C7	1.388 (3)	O3W—H3B	0.82 (3)
			
O1W^i^—Cu1—O1W	180.00 (7)	C5—C6—H6	120.4
O1W^i^—Cu1—N2	89.17 (8)	C7—C6—H6	120.4
O1W—Cu1—N2	90.83 (8)	C6—C7—C2	121.0 (2)
O1W^i^—Cu1—N2^i^	90.83 (8)	C6—C7—H7	119.5
O2W—Cu1—N2	95.22 (7)	C2—C7—H7	119.5
O2W—Cu1—O1W^i^	87.99 (7)	N2—C8—N1	109.97 (19)
O2W—Cu1—N2^i^	84.78 (7)	N2—C8—H8	125.0
N2—Cu1—N2^i^	180.0	N1—C8—H8	125.0
O1—C1—O2	125.1 (2)	N3—C9—N1	111.1 (2)
O1—C1—C2	118.90 (19)	N3—C9—H9	124.4
O2—C1—C2	115.97 (19)	N1—C9—H9	124.4
C3—C2—C7	118.7 (2)	C8—N1—C9	104.65 (18)
C3—C2—C1	120.38 (19)	C8—N1—C5	128.54 (18)
C7—C2—C1	120.9 (2)	C9—N1—C5	126.81 (18)
C4—C3—C2	121.1 (2)	C8—N2—N3	107.78 (17)
C4—C3—H3	119.4	C8—N2—Cu1	133.37 (15)
C2—C3—H3	119.4	N3—N2—Cu1	117.25 (13)
C3—C4—C5	119.1 (2)	C9—N3—N2	106.47 (18)
C3—C4—H4	120.5	Cu1—O1W—H1A	107.7 (19)
C5—C4—H4	120.5	Cu1—O1W—H1B	109 (2)
C6—C5—C4	121.0 (2)	H1A—O1W—H1B	102 (3)
C6—C5—N1	120.03 (19)	H2A—O2W—H2B	107 (3)
C4—C5—N1	118.95 (19)	H3A—O3W—H3B	111 (3)
C5—C6—C7	119.2 (2)		
			
O1—C1—C2—C3	164.7 (2)	N3—C9—N1—C8	0.0 (3)
O2—C1—C2—C3	−16.6 (3)	N3—C9—N1—C5	179.9 (2)
O1—C1—C2—C7	−17.6 (3)	C6—C5—N1—C8	8.8 (3)
O2—C1—C2—C7	161.1 (2)	C4—C5—N1—C8	−171.9 (2)
C7—C2—C3—C4	−0.7 (3)	C6—C5—N1—C9	−171.1 (2)
C1—C2—C3—C4	177.1 (2)	C4—C5—N1—C9	8.1 (3)
C2—C3—C4—C5	0.0 (3)	N1—C8—N2—N3	−0.7 (3)
C3—C4—C5—C6	0.6 (3)	N1—C8—N2—Cu1	163.97 (15)
C3—C4—C5—N1	−178.58 (19)	O1W^i^—Cu1—N2—C8	167.8 (2)
C4—C5—C6—C7	−0.6 (3)	O1W—Cu1—N2—C8	−12.2 (2)
N1—C5—C6—C7	178.65 (19)	O1W^i^—Cu1—N2—N3	−28.63 (17)
C5—C6—C7—C2	−0.2 (3)	O1W—Cu1—N2—N3	151.37 (17)
C3—C2—C7—C6	0.8 (3)	N1—C9—N3—N2	−0.4 (3)
C1—C2—C7—C6	−177.00 (19)	C8—N2—N3—C9	0.7 (3)
N2—C8—N1—C9	0.5 (3)	Cu1—N2—N3—C9	−166.87 (17)
N2—C8—N1—C5	−179.47 (19)		

Symmetry codes: (i) −*x*, −*y*, −*z*+2.

### Hydrogen-bond geometry (Å, °)

**Table:**

*D*—H···*A*	*D*—H	H···*A*	*D*···*A*	*D*—H···*A*
O1W—H1A···O2^ii^	0.87 (2)	2.12 (2)	2.948 (3)	158 (2)
O1W—H1B···N3^i^	0.87 (2)	2.27 (3)	2.873 (3)	126 (3)
O2W—H2A···O1^iii^	0.82 (3)	1.98 (3)	2.794 (2)	172 (3)
O2W—H2B···O2^iv^	0.82 (3)	1.91 (3)	2.711 (2)	167 (3)
O3W—H3A···O2^ii^	0.84 (3)	1.97 (3)	2.789 (2)	167 (3)
O3W—H3B···O2W^v^	0.82 (3)	1.94 (3)	2.758 (2)	170 (3)

Symmetry codes: (ii) −*x*+1, −*y*+1, −*z*+1; (i) −*x*, −*y*, −*z*+2; (iii) *x*−1, *y*−1, *z*+1; (iv) −*x*, −*y*+1, −*z*+1; (v) −*x*, −*y*+1, −*z*+2.
